# A practical evidence-based approach to management of type 2 diabetes in children and young people (CYP): UK consensus

**DOI:** 10.1186/s12916-024-03349-4

**Published:** 2024-04-02

**Authors:** Billy White, S. M. Ng, J. C. Agwu, T. G. Barrett, N. Birchmore, M. Kershaw, J. Drew, F. Kavvoura, J. Law, C. Moudiotis, E. Procter, P. Paul, F. Regan, P. Reilly, P. Sachdev, R. Sakremath, C. Semple, K. Sharples, M. Skae, A. Timmis, E. Williams, N. Wright, A. Soni

**Affiliations:** 1 University College London Hospitals NHS Foundation Trust, London, UK; 2 Mersey And West Lancashire Teaching Hospitals NHS Trust, Ormskirk, UK; 3 Wye Valley NHS Trust, Hereford, UK; 4 Birmingham Women’s And Children NHS Foundation Trust, Birmingham, UK; 5 Great Ormond Street Hospital For Children, NHS Foundation Trust, London, UK; 6 Nottingham University Hospitals NHS Foundation Trust, Nottingham, UK; 7 Royal Berkshire NHS Foundation Trust, Reading, UK; 8 Royal Devon and Exeter NHS Foundation Trust, Exeter, UK; 9 Alder Hey Children’s NHS Foundation Trust, Liverpool, UK; 10 Guy’s and St Thomas’s NHS Foundation Trust, London, UK; 11 Torbay and South Devon NHS Foundation Trust, Torquay, UK; 12 Shrewsbury and Telford Hospital NHS Trust, Shrewsbury, UK; 13 University Hospitals Bristol and Weston NHS Foundation Trust, Bristol, UK; 14 Bart’s Health NHS Trust, London, UK; 15 Central Manchester University Hospitals NHS Foundation Trust, Manchester, UK; 16 Countess of Chester Hospital NHS Foundation Trust, Chester, UK; 17 Hampshire Hospitals NHS Foundation Trust, Winchester, UK; 18 Sheffield Children’s Hospital NHS Foundation Trust, Sheffield, S102TH UK

**Keywords:** Type 2 diabetes, Children and young people, Complications of excess weight, Metformin, Physical activity, Bariatric surgery

## Abstract

**Background:**

Type 2 diabetes in young people is an aggressive disease with a greater risk of complications leading to increased morbidity and mortality during the most productive years of life. Prevalence in the UK and globally is rising yet experience in managing this condition is limited. There are no consensus guidelines in the UK for the assessment and management of paediatric type 2 diabetes.

**Methods:**

Multidisciplinary professionals from The Association of Children’s Diabetes Clinicians (ACDC) and the National Type 2 Diabetes Working Group reviewed the evidence base and made recommendations using the Grading Of Recommendations, Assessment, Development and Evaluation (GRADE) methodology.

**Results and discussion:**

Young people with type 2 diabetes should be managed within a paediatric diabetes team with close working with adult diabetes specialists, primary care and other paediatric specialties. Diagnosis of diabetes type can be challenging with many overlapping features. Diabetes antibodies may be needed to aid diagnosis. Co-morbidities and complications are frequently present at diagnosis and should be managed holistically. Lifestyle change and metformin are the mainstay of early treatment, with some needing additional basal insulin. GLP1 agonists should be used as second-line agents once early ketosis and symptoms are controlled. Glycaemic control improves microvascular but not cardiovascular risk. Reduction in excess adiposity, smoking prevention, increased physical activity and reduction of hypertension and dyslipidaemia are essential to reduce major adverse cardiovascular events.

**Conclusions:**

This evidence-based guideline aims to provide a practical approach in managing this condition in the UK.

## Background

Type 2 diabetes (T2DM) in young people is an aggressive disease with a greater risk of complications leading to increased morbidity and mortality during the most productive years of life [1]. Evidence and experience are limited in this age group, with much of the data coming from observational studies such as SEARCH [2], the TODAY randomised control study and more recently GLP1 drug studies [3].

These guidelines aim to improve the care of children and young people (CYP) in the UK with type 2 diabetes. Most paediatric diabetes multi-disciplinary teams have good experience in managing type 1 diabetes and complex conditions yet relatively little experience managing type 2 diabetes and associated co-morbidities.

## Methods

The Association of Children’s Diabetes Clinicians (ACDC) and National Type 2 Diabetes Working Group co-established a guideline development group, which included a multidisciplinary group of health professionals including general paediatricians, paediatric endocrinologists, diabetes specialist nurses, dietitians and psychologists. The group members agreed on the scope and subdivided topics based on areas of interest and expertise. The subgroups met independently to review the evidence base. The whole group met via teleconference over 1 year to review the evidence and make recommendations. Grading was undertaken using GRADE (Grading Of Recommendations, Assessment, Development and Evaluation).

The guideline was reviewed and endorsed by the Association of British Clinical Diabetologists (ABCD), ACDC and the British Society of Paediatric Endocrinology and Diabetes (BSPED) (Table 1).

**Table 1:** Summary of the recommendations with GRADE methodology

Sections	Recommendations	GRADE
Screening and diagnosis	Asymptomatic screening is recommended if there is a high body mass index (BMI > 85th centile) and 1 or more of: first- or second-degree family history of type 2 diabetes, high-risk race/ethnicity, gestational diabetes or insulin resistance (acanthosis nigricans or presence of other metabolic conditions, such as hypertension and hyperlipidaemia, PCOS or SGA)	B
	If a diagnosis is suspected based on clinical symptoms, then a point of care random blood glucose must be performed on the same day and abnormal results discussed with the local diabetes team via locally agreed pathways	D
	Diagnosis can be made based on fasting glucose, or 2-h glucose concentration during an oral glucose tolerance test (OGTT) or HbA1c. Any of the following are diagnostic of diabetes:	B
	• HbA1c ≥ 48 mmol/mol (6.5%). Must utilise a laboratory-based, DCCT-aligned, National Glyco-haemoglobin Standardization Program (NGSP) certified methodology that is validated for diagnosis. Do not use HbA1c in patients with haemoglobinopathies, within 3 months of transfusion or with increased red cell turnover	
	• Fasting plasma glucose (FPG) ≥ 7.0 mmoL/L (126 mg/dL)	
	• Post OGTT 2-h plasma glucose ≥ 11.1 mmoL/L (200 mg/dL), with 1.75 g/kg (max 75 g) anhydrous glucose dissolved in water	
	• Symptoms of diabetes(including polyuria, polydipsia and unexplained weight loss) and a random plasma glucose > 11.1 mmol/L (200 mg/dL)	
	• In the absence of symptoms, hyperglycaemia detected incidentally or under conditions of acute physiologic stress may be transitory and should not be regarded as diagnostic of diabetes. Diagnosis should be confirmed with repeat testing on a different day	
	OGTT need not be done routinely where HbA1c is ≥ 48 mmol/mol (6.5%) and should not be done where HbA1c is < 6.0% (42 mmol/mol). Those with HbA1c 42–48 mmol/mol (6–6.5%) should be evaluated clinically and clinical judgement used to decide between a repeat HbA1c in 3–6 months or an OGTT	GP
	HbA1c 42–48 mmol/mol (6–6.5%) is diagnostic of pre-diabetes, the management of this is outside the scope of this guideline	B
	Once diabetes is diagnosed, the type of diabetes should be diagnosed clinically based on signs and symptoms, biochemical investigations, family history and clinical progression. Monogenic diabetes must be considered, especially where the clinical picture is atypical for type 1 or 2. Where there is diagnostic doubt, the patient should receive appropriate safety netting for the possibility of evolving type 1 diabetes (including blood ketone testing) and be re-evaluated after a short period of time	GP
	Diabetes autoantibody testing (GAD, IA2 and ZnT8) should be considered in paediatric patients with the clinical diagnosis of T2DM because of the high frequency of islet cell autoimmunity in otherwise “typical” T2DM and where there is diagnostic uncertainty for example where symptomatic or those with lean body weight. Those with diabetes antibodies are more likely to need insulin early	B
Glycaemic targets	HbA1c should be measured every 3 months in CYP with T2DM	GP
	HbA1c targets should be individualised, noting the low risk of hypoglycaemia	E
	Intensify treatment if HbA1c not below 48 mmol/mol (6.5%) at 3 months	B
	Individualise HbA1c Target after the first 3 months but aim to keep below 48mmol/mol (6.5%)	E
Self-monitoring of blood glucose	All patients should be taught to self-manage blood glucose (SMBG) and have the necessary equipment provided	GP
	Frequency of SMBG for CYP with T2DM on basal insulin should be individualised including taking into account the risk of hypoglycaemia, glycaemic target and stage of treatment	GP
	Those not on insulin should be encouraged to test several times a week consisting of both fasting and post-prandial levels to monitor disease progression, provide data for clinical consultations and predict glycaemic control. Testing may be more intense initially and during periods of changing blood glucose levels (e.g. illness or fasting) but may reduce when levels are stable for several weeks	GP
	Those on multiple daily injections of insulin should undertake SMBG at least 5 times a day to adjust doses and monitor for hypoglycaemia, similar to CYP with T1DM on MDI	GP
	Fasting, pre-prandial and post-prandial blood glucose level targets should be set to allow patients to monitor their progress. Fasting and pre-prandial levels should ideally be 3–7 mmol/L (3.9–7 mmol/L if on insulin) and post-prandial should aim to be < 10 mmol/L	GP
Continuous glucose monitoring	There is no evidence available to routinely recommend the use of CGMS or FGS in children and young people with type 2 diabetes. Individualised considerations such as learning difficulties or needle phobia may influence management. Brief periods of CGMS/FGS may be indicated during treatment intensification or education	GP
Structured education	Individualised Structured diabetes education should be provided to CYP with T2DM and their carers at the time of diagnosis, revised soon after diagnosis then annually or more frequently dependent on individual need	GP
	No single structured programme is recommended, with TSDE, Be Healthy For Life and iCAN being examples of existing programmes. (http://main.diabetes.org/dorg/PDFs/Type-2-Diabetes-in-Youth/Type-2-Diabetes-in-Youth.pdf). (portal.bsc.gwu.edu/web/today)	C
MDT approach	MDT approach (including dietitian, specialist nurse and psychologist support) to T2DM management in children is key to improving outcomes	D
	A unified approach with consistent team messaging improves outcomes	D
	T2DM should be managed in the secondary care setting with close integration with primary care. GP primary health team members should be involved in the child’s care in line with the whole family approach, and additional resources such as social prescribing and exercise prescription	D
	Multi-agency working is important to be included in the MDT approach (such a youth support workers, family support workers, patient advocate, schools, school nurses, social workers)	D
	Telemedicine could help establish regular contact with the MDT in T2DM patients and their families (including home health monitoring, video clinics, telephone, emails and text support) whilst acknowledging that access may be challenging (including digital poverty)	D
	Collaboration with an adult diabetes MDT team is essential (ideally young adult service) to enable seamless transition and support with complex cases	GP
Weight loss	Weight reduction as part of a strategy to reduce BMI should form part of diabetes management in youth with T2DM as this has been associated with better glycaemic outcomes (Grade C)	C
	CYP with T2DM should be offered a family-based weight loss programme based on a range of personalised diet strategies, physical activity and behavioural intervention to treat obesity (Grade B)	D
	There is currently insufficient evidence in CYP to recommend specific targets for weight loss to control or reverse T2DM	D
	• Targets should be at minimum in line with accepted recommendations for the management of obesity. It is important to remember that increasing height in pre-pubertal and pubertal young people results in a reduction in BMI. Targets for weight should be set to reduce weight by 5% in the first 3 months in pre-pubertal and pubertal CYP	
	• Post-pubertal young people should be advised to aim for 5% weight loss in 3–6 months and 10% weight loss in the 1st year	
	• In the longer term, CYP should be encouraged to aim to reduce BMI to below 85th centile. Professionals need to be aware of this	
Dietary Modifications	An individualised and family-wide approach to dietary modification is essential. A specialised paediatric diabetes dietitian should work together with the family to identify potential diet and lifestyle changes and formulate a plan with SMART (specific, measurable, achievable, relevant, time bound) goals [4–6]	B
	Regular follow-up appointments with a specialised paediatric diabetes dietitian are essential to monitor progress and review goals. Additional appointments with the specialised paediatric diabetes dietitian outside the MDT every 4–8 weeks should be considered	GP
	Families should be encouraged to follow a healthy balanced diet that is rich in wholegrains, vegetables, fruit, low-fat dairy, nuts and seeds and limits fats, oils and sugary foods [7–14]	B
	Families should be encouraged to consume balanced meals including complex carbohydrate, protein and vegetables to regulate appetite and improve satiety after a meal [15–18]	B
	Intake of sugary drinks should be limited [19–23]	C
	If there has been no significant weight loss 6 months after diagnosis while aiming to implement a healthy balanced diet, then alternative approaches can be considered. There is limited evidence in paediatrics that one particular dietary intervention is more successful than another therefore evidence has been extrapolated from adult data. One of the following approaches to promote weight loss can be considered:	
	• A healthy low-fat diet (limits fat to 30% of energy intake, 45–50% of energy from carbohydrates and 20–25% of energy from protein) [9, 24]	B
	• A lower carbohydrate diet(limits carbohydrate to 30% of energy intake,45% of energy from fat and 20% of energy from protein) [25]	D
	• A low-energy diet using either meal replacement products or a guided amount of calories from food at each mealtime that limits energy intake to 800kcal a day for an 8–12 week period (post-pubertal, individualised, adult data). This initial phase is followed by the gradual reintroduction of a healthy balanced diet. This may be a suitable treatment option for young people who are keen to follow a structured plan. Careful consideration should be given to the appropriateness of this option and take in to account life events such as exams, learning to drive and family celebrations [10, 26–30]	C
Physical Activity	Physical activity levels recommended for CYP with T2DM are the same as for young people without T2DM across countries (UK, USA, Europe) and the suggestion is for 60 min per day of moderate/vigorous physical activity to improve body composition and insulin sensitivity	B
	For CYP with T2DM, exercise may need to occur in more than one session a day or start with a lesser amount and build up as able	GP
Psychological management	CYP must have on-going access to mental health professionals embedded within a diabetes team	GP
	All CYP should be screened for mental health regularly by a team member (at least annually, or more frequently with inadequate diabetes control) including depression and eating disorders	GP
	• The PedsQLTM Diabetes Module is a specific module of the PedsQLTM freely available for clinical use through registration—https://eprovide.mapi-trust.org/instruments/pediatric-quality-of-life-inventory. It effectively screens for diabetes-related quality of life. PHQ-2 is used for screening for depression	
	• There is no suitable validated eating disorder questionnaire that adequately screens for binge eating in this population. However, the Adolescent Binge Eating Disorder Questionnaire ADO-BED has been validated in young people with obesity to identify those at risk of binge eating disorder (https://www.medicalhomeportal.org/link/6884)	
Pharmacotherapy	Metformin monotherapy is effective in achieving metabolic control in youth with T2DM at least initially after diagnosis	A
	Initial treatment with insulin ± metformin based on metabolic state and ketosis. Metabolically unstable CYP with diabetes being started on s/c or IV insulin	D
	Once daily basal insulin with a starting dose of 0.25–0.5 units/kg/day. Evidence for HbA1c cut off 69 mmol/mol (8.5%) Maximum dose of basal insulin 1.5 units/kg	C
	Weaning off insulin after initial treatment	A
	Evidence for HbA1c cut off 69 mmol/mol (8.5%)	C
	Addition of insulin if metformin monotherapy fails and beta cell loss	A
	Prandial insulin and dose	E
	Liraglutide as an adjunct	B
Bariatric surgery	Bariatric surgery in adolescents with BMI > 35 kg/m^2^ shows clear benefit in remitting T2DM	A
	Surgery should be considered as an option for treatment of obesity-related T2DM for obese CYP who are demonstrating inadequate response to pharmacological treatments within 12–18 months to avoid a reduction in beta-cell mass	GP
	Bariatric surgery is effective in inducing remission and reducing the progression in diabetic nephropathy and improving hypertension	C
	Bariatric surgery has been demonstrated to stabilise the progression of diabetic retinopathy (DR) but evidence for long-term resolution of DR is currently not available	D
	Monitoring of long-term complications post-bariatric surgery in CYPs is required for nutritional deficiencies and metabolic bone health	B
	There is inadequate data to support bariatric surgery in prepubertal children with obesity-related T2DM with limited data on long-term outcomes	GP
Hypertension	Blood pressure should be measured, using an appropriately sized cuff, at every clinic appointment — at least four times per year	D
	Blood pressure readings should be interpreted using a paediatric blood pressure centile chart	B
	Hypertension is defined as systolic or diastolic pressure equal to or greater than the 95th centile for age and sex on 3 separate occasions. If there is any concern about transient, stress-related, high blood pressure readings (white coat syndrome) ambulatory blood pressure monitoring should be considered	B
	Increased BMI is a risk factor for developing hypertension and should be measured at every clinic appointment	B
	Once a diagnosis of hypertension has been made initial treatment should focus on weight reduction, exercise and reducing salt in-take [31]	C
	If after 6 months blood pressure is still above the 95th centile for age, consider starting an ACE inhibitor [32]—the teratogenic risks of ACE inhibitors should be discussed with female adolescents and steps advised to mitigate against these risks, before starting treatment	
	• ACE inhibitors are particularly beneficial in young people with diabetes due to their reno-protective effect. Take advice from an adult diabetologist/paediatric nephrologist regarding other options	B
	• For the Afro-Caribbean population, consideration should be given to other causes of hypertension (refer to hypertension guidelines) but it is likely to be due to metabolic syndrome	
	• The more common side effects of ACE inhibitors—abnormalities in sodium and potassium can be mitigated against by monitoring electrolytes with a blood test before starting treatment and at 4–6 weeks afterwards. Hypotension—consider a test dose with pre- and post-blood pressure monitoring on a paediatric assessment unit before starting regular treatment	D
	The aim of treatment should be to reduce blood pressure to less than the 90th centile for age, height and gender	B
	If ACE inhibitors are not tolerated (the most common side effect, is a dry cough) angiotensin receptor blocker should be used [31]	D
	If, despite treatment, blood pressure is not lowered below the 90th centile referral to a tertiary hypertension service would be appropriate [31]	D
Lipids	Lipid testing should be performed when initial glycaemic control has been achieved or after 3 months of treatment, and annually thereafter	B
	The initial screening lipid profile does not need to be a fasted sample. Use non-HDL-C level for initial screening	A
	Lipid targets are:	
	◦ LDL-C < 2.6 mmol/L (100 mg/dL)	
	◦ HDL-C > 0.91 mmol/L( 35 mg/dL)	
	◦ Triglycerides < 1.7 mmol/L(150 mg/dl)	
	◦ Non HDL-C < 3.6 mmol/L (140 mg/dL)	
	If abnormal profile, initial treatment is by diet modification and improved glycaemic control	A
	• Limit calories from total fat to 25–30%	
	• Limit saturated fat to < 7%	
	• Limit cholesterol to < 200 mg/day	
	• Avoid trans fats	
	• For elevated LDL, aim for about 10% calories from monounsaturated fats	
	• For elevated triglycerides, decrease simple sugar intake and increase dietary n-3 fatty acids in addition to the above changes. (A) [33]	
	A repeat lipid profile should be performed at 6 months following the dietary intervention and weight management (fasted)	B
	If repeat fasting LDL-C remains > 3.4 mmol/L (130 mg/dL), treatment with a statin should be commenced. The ideal treatment target is to reduce the LDL-C to < 2.6 mmol/L (100 mg/dL)	B
	Statins can be used in children over the age of 10 years with type 2 diabetes [34–36]	A
	• High-intensity statins are recommended for the treatment and prevention of cardiovascular disease in patients with T2D. Atorvastatin and rosuvastatin are recommended as can achieve a high-intensity LDL-C lowering effect (> 40%) at lower doses than other statins. They can be prescribed in CYP 10–17 years old (BNFc)	
	• Statins should be started at the lowest available dosage (atorvastatin 10 mg daily, rosuvastatin 5 mg daily) and increased based on response and side effects	
	• If LDL-C target levels are not achieved with at least 3 months of compliant use the dose should be increased by 1 increment (usually 10 mg). The dose can be increased by a further increment after a further 3 months or a second agent could be added under the guidance of a lipid specialist (e.g. bile acid sequestrant or cholesterol absorption inhibitor)	B
	• Liver enzymes should be measured before treatment and repeated within 3 months and at 12 months of starting treatment	
	• Statins should be used with caution in patients at increased risk of muscle toxicity, including those with a personal or family history of muscular disorders, previous history of muscular toxicity, a high alcohol intake, renal impairment or hypothyroidism. Creatinine kinase concentration should be measured in children before treatment and if unexplained muscle pain occurs. Hypothyroidism should be treated before starting statin treatment. (NICE, BNFc)	B
	• Statins are teratogenic and contraindicated in pregnancy — caution with use in adolescent girls	
	If fasting triglycerides are > 5.6 mmol/L (400 mg/dL) or > 11.3 mmol/L (> 1000 mg/dL) non-fasting, there is a risk of developing pancreatitis	C
	Fibrates can be used in children over the age of 10 years for significant hypertriglyceridaemia	A
	There are no good RCTs to support the use of omega-3 or fish oils in children or adolescents [37]. Studies have shown benefits in adults in reducing triglyceride levels	A
	Recommend smoking cessation	B
NAFLD	Initial screening with ALT at diagnosis and annually thereafter if normal results	B
	Investigate for NAFLD/NASH and other causes of liver disease if persistently raised ALT (> 2 × ULN) as ALT not specific for NAFLD	C
	Consider referral to paediatric hepatology/gastroenterology if ALT levels > 3 × ULN despite dietary modifications and improved glycaemic control	A
	General dietary and lifestyle advice aiming to achieve weight reduction is important however there are currently no specific dietary recommendations shown to reverse or slow the progression of NAFLD	A
	Management of extrahepatic co-morbidities known to accelerate NAFLD ie insulin resistance and dyslipidaemia is important; however, there are no currently recommended pharmacological treatments specifically for treating NAFLD in children	B
	Assessment and treatment of co-morbidity (obstructive sleep apnoea, vit D deficiency)	GP
	Psychological support is important	GP
Retinopathy	Diabetic retinopathy screening should start from the age of 12 and annually thereafter, in line with ace advice	B
	Improvement in glycaemic control remains the cornerstone of primary prevention and also mitigates progression once retinopathy has developed	B
	Adult literature recommends treatment of hypertension and dyslipidaemia in the presence of diabetic retinopathy	B
	Sudden intensification of glycaemic treatment with a rapid reduction in HbA1c may lead to a rapid progression of retinopathy	B
Micro-albuminaemia	Monitoring for albuminuria should commence at the time of diagnosis and annually thereafter by using the early morning spot urine (EMU) for albumin to creatinine ratio (ACR)	B
	An elevated urine ACR of above 3 mg/mmol, should be confirmed by repeating the test on 2 further occasions on different days within a 3- to 6-month period	B
	For moderately elevated albuminuria (Urine ACR 3–30 mg/mmol), a multidisciplinary team approach should be applied to improve glycaemic control along with targeting other risk factors(smoking, obesity and hypertension) for albuminuria to improve the outcome	B
	Use an ACE-inhibitor (ACE-I) or angiotensin receptor blocker (ARB), in non-pregnant patients with type 2 diabetes in the following circumstances:	B
	• For those with moderately increased albuminuria (3–30 mg/mmol)	
	• For those with severely increased albuminuria (> 30 mg/mmol)	
	Referral to specialist renal/Kidney care services is recommended for children and young person (CYP) with chronic kidney disease (CKD) in the following circumstances:	B
	• GFR < 30 ml/min/1.73 m^2^ and/or consistent finding of significant albuminuria (> 30 mg/mmol) or albumin excretion rate (AER) > 300 mg/24 h	
	• Significant haematuria	
	• Renal structural abnormality on ultrasound	
Obstructive sleep apnoea	The gold standard diagnostic test is overnight laboratory PSG, which allows for the quantification of episodes of apnoea and hypopnoea per hour of sleep	B
	Clinical signs and symptoms are not always specific. A validated sleep questionnaire may be helpful. Clinicians should have a high index of suspicion for SA	B
	If symptoms and home sleep monitoring devices are suggestive, the diagnosis of OSA should be made by formal polysomnography and referral to sleep specialist for further management	B

## Results and discussion

### Diagnosis and investigations

The main risk factors for developing T2DM are excess weight, first or second-degree relative with T2DM, maternal gestational diabetes, high-risk race/ethnicity and insulin resistance. Insulin resistance is hard to measure but associated with acanthosis nigricans, early poor growth (small for gestational age) and other associated comorbidities such as hypertension, hyperlipidaemia and polycystic ovary syndrome (PCOS).

We recommend HbA1c testing as the primary test for screening for type 2 diabetes. The choice of screening test is controversial with HbA1c increasingly recommended over the gold standard oral glucose tolerance test despite limited evidence in CYP. HbA1c does not require a fasting sample and is a predictor of vascular disease across a wider glycaemic range than just the diabetic one. However, it lacks sensitivity and would miss some people with diabetes. Testing fasting blood glucose in addition to HbA1c may diagnose some additional patients but may not be pragmatic in those unlikely to return for a fasted sample. The oral glucose tolerance test is more sensitive, but is inconvenient, more costly, has imperfect reproducibility and is less popular, contributing to poorer uptake [38, 39].

Identification of diabetes type can be challenging with all types having overlapping features, particularly in those with excess weight [1]. Detection of diabetes antibodies identifies those at the highest risk of developing auto-immune driven loss of insulin production and can aid with the correct identification of diabetes subtype. There is insufficient evidence for the use of insulin or C-peptide levels at diagnosis.

### Glycaemic targets

Intensive glucose control in adults improved HbA1c and any diabetes-related end point but not mortality [40, 41]. No similar robust studies exist for CYP.

TODAY showed that an HbA1c of 6.3% (45 mmol/mol) or more after initiation of metformin was a predictor of eventual loss of glycaemic control irrespective of treatment arm (defined as HbA1c > 8% (64 mmol/mol)). with every 0.1% increase in HbA1c increasing risk by 16%, with a median time of approximately 11 months to loss of control [42].

We recommend that HbA1c is tested every 3 months with an overall target of < 6.5% (48 mmol/mol) which should be individualised based on circumstances.

### Self-monitoring of blood glucose (SMBG)

There is no high-quality evidence that self-monitoring of blood glucose (SMBG) in CYP improves outcomes or that more intense monitoring is better. Overall adherence with twice daily SMBG was low in TODAY (59% initially, < 50% by 12 m) with greater use associated with lower HbA1c [43]. Similar findings were seen in those using insulin in SEARCH [44]. No association was found between SMBG and quality of life (QOL) or depression; however, there remains a significant financial and personal burden in monitoring without clear evidence of benefit [43].

We recommend that self-monitoring of blood glucose is undertaken in all patients in line with other international guidelines [45–47]. Monitoring can guide management where the diabetes subtype is not clear and where disease progression is more aggressive or rapid. Frequency should be individually tailored based on hypoglycaemia risk, availability, burden and need for treatment adjustment.

### CGM and FGM

There is inadequate evidence to guide the use of continuous glucose monitoring (CGM) or flash glucose monitoring (FGM) in CYP. Inconsistent results are seen in adults [48, 49] and there is a lack of data in CYP with type 2 diabetes. Further research is needed to identify which patients may benefit from the use of CGM and FGM and how they should be used to maximise patient benefit and cost-effectiveness.

There may be a role for the use of CGM/FGM in certain circumstances such as learning difficulties, insulin usage or as a short-term intervention during treatment intensification and education [46].

### Structured education

Diabetes-structured education programmes first started in the 1970s. The focus has changed over time from a knowledge-based approach to one supporting self-empowerment. Meta-analysis of structured programmes for CYP with type 1 diabetes found no benefit of structured education versus over informal unstructured education in improving glycaemic control [50]. Similar findings were seen in adults with type 2 diabetes [51].

Two structured education programmes have been developed for CYP with type 2 diabetes but neither have undergone RCT evaluation: the TODAY Standard Education Diabetes Education (TSDE) and iCAN [52, 53]. The iCAN programme is a bespoke group intervention delivered over four 2-h workshops with a focus on food, activity and emotional well-being [53].

### Multidisciplinary team (MDT) approach

Multi-disciplinary diabetes team structure and approach recommendations are based on observational studies and expert opinion [45, 54, 55]. We recommend that CYP should be managed by multi-disciplinary secondary care teams with close integration with primary care and collaboration with adult diabetes teams. Teams should include dietitians, paediatric nurses, diabetes educators, psychologists, social workers (sometimes called community/cultural workers) and medical doctors. Multi-agency working is important, including links with youth, family and social workers, and schools.

A unified approach with consistent team messaging likely improves outcomes. Telemedicine may improve access but access can be challenging (including digital poverty).

### Lifestyle interventions in type 2 diabetes management in children

Lifestyle change is the cornerstone of type 2 diabetes management.

### Weight loss targets

Weight loss in adults improves glycaemic control, with clinical benefits seen from 5% loss and further improvements with additional loss [56–58]. There is no direct evidence in young people with type 2 diabetes to recommend a target weight loss as no lifestyle RCT has aimed for sufficient energy restriction to result in 5–10% weight loss seen in adult studies.

Good diabetes outcomes are not dependent on weight loss. The highest improvement of glycaemic control in the TODAY study was seen in the group receiving rosiglitazone even though that group had the greatest increase in body mass index (BMI). However, weight loss of > 7% across all treatment groups was associated with small benefits in cardiometabolic risk factors [59].

### Dietary modifications

Dietary recommendations are based on healthy eating principles for all young people and families. A healthy balanced diet is considered to be rich in wholegrains, vegetables, fruit, dairy, nuts and seeds and limits fats, oils and sugary foods. Carbohydrates should provide 40–50% of energy requirements, fat < 35% and protein 15–25%. There is little evidence to suggest other macronutrient balances at the population level; however, the ratio of carbohydrates to fat can be individualised if standard advice does not promote weight loss. Some may benefit from lower carbohydrate intake (and higher fat) whilst others benefit from lower fat.

A whole-family approach should be used to enable change across the whole family with individualisation for each family. Support from specialist diabetes dietitians is likely to be needed and should be offered to all families.

### Physical activity

Physical activity is likely to have multiple beneficial health benefits however the evidence for improvement in glycaemic control in youth is limited [60]. A 2010 systematic review found no evidence to guide physical activity modification [61] and the addition of intensive lifestyle support in TODAY improved weight but not glycaemic control [62].

Multiple cohorts show that young people with type 2 diabetes undertake insufficient physical activity: in one cohort 55.7% undertook no regular physical activity, [63] and in another only 6.5 min of moderate/vigorous activity was undertaken each day [64].

Most national guidelines recommend 60 min per day of moderate to vigorous exercise and these should also apply to those with type 2 diabetes [65].

### Psychological management

Youth with type 2 diabetes have a higher prevalence of moderate or severe depression than those with type 1 (18 vs. 5% in boys and 20 vs. 9% in girls, respectively), with higher mean HbA1c and frequency of emergency department visits associated with depressed mood [66].

Over 25% reported symptoms of disordered eating behaviours in SEARCH and TODAY, such as skipping insulin, vomiting, and using diet pills or laxatives, and these behaviours were associated with poorer glycaemic control in females [67] and more severe obesity, psychological symptoms of disordered eating, and symptoms of depression [68].

Addressing psychological needs should be incorporated within everyday practice. The PedsQL (paediatric quality of life) questionnaire can be used to evaluate and monitor ongoing issues [69]. There is limited research to determine which behavioural interventions are most effective [4] and approaches could include personalised tailored interventions to their interests, health coaching and behaviour therapy and the use of peer counsellors. These are likely to need to address cultural differences and home lifestyles in order to achieve better outcomes [5].

### Pharmacotherapy

#### Initial treatment

Initial treatment of a CYP with obesity and diabetes should take into account that diabetes type is relatively uncertain for the first few weeks, due to overlap in presenting symptoms and a significant number presenting with ketosis [6].

Metformin should be started once any ketosis has resolved, starting at a low dose to minimise side-effects and titrated to a maximum of 2 g per day, or the maximum tolerated dose. Sustained release preparations are available and should be considered if there are GI side effects or compliance issues that could improve with reduced dose frequency [7].

Patients who present with ketosis, polydipsia, polyuria, weight loss, or have HbA1c > 8.5% (69.4 mmol/mol) should also be treated with insulin. This should be with intravenous insulin if unwell (e.g. systemic illness, not tolerating food or drink), and then subcutaneous basal insulin at 0.25–0.5 units/kg/day titrated to a maximum of 1.5 units/kg/day, pending antibody results and improved glycaemia.

There is some evidence that insulin can be successfully weaned off after initially starting it [8]. Basal insulin can be tapered over 2–6 weeks by decreasing the insulin dose by 10–30% each time the metformin is titrated up and further once metformin is at the maximum dose [9]. There is no evidence that continuing insulin will preserve B-cell function [10].

#### Treatment Intensification

The goal of initial treatment should be to attain an HbA1c of less than 6.5% (48 mmol/mol).

##### Liraglutide

Failure to achieve goal HbA1c with metformin monotherapy should prompt consideration of second-line treatment with liraglutide. Liraglutide is licensed for those aged 10 years and above with a BMI > 85th centile.

Liraglutide is given as daily subcutaneous injections and can be started at 0.6 mg daily and can be increased with 0.6 mg increments every 1–2 weeks up to 1.8 mg daily based on fasting capillary blood glucose > 6 mmol/L and tolerability.

Side effects are mainly gastrointestinal (nausea and diarrhoea). Acute pancreatitis is a rare and theoretical risk and patients should be counselled and monitored for signs including persistent, severe abdominal pain. Monitoring of pancreatic amylase and lipase should occur at baseline, after the first clinical review and yearly thereafter. If pancreatitis is suspected, liraglutide should be stopped and reported to the medicines and health products regulatory agency (MHRA) via the Yellow Card scheme.

##### Insulin

Basal insulin therapy is the only remaining licensed treatment option if metformin and liraglutide are insufficient or not tolerated. Insulin resistance is characteristic in CYP going through mid-late puberty and might require higher doses of basal insulin up to 1.5 units/kg/day to achieve glycaemic control. A single daily long-acting insulin analogue (e.g. glargine, detemir or degludec) is preferred as studies show that compliance with insulin can be a challenge in CYP [11, 12]. Higher concentrations of basal insulin (U-300 glargine, U-200 degludec) may be required to avoid large-volume injections that may further diminish medication adherence [46].

Additional meal-time rapid-acting insulin (e.g. aspart) may be needed if basal insulin cannot achieve glycaemic control. Doses should be titrated based on pre- and post-prandial readings and should be done in discussion with a dietitian who is supporting the young person on lifestyle changes, including diet and weight management.

The main side effects of insulin treatment are hypoglycaemia and weight gain. The incidence of hypoglycaemia is low however all patients should be educated on its treatment, including the use of glucagon [42].

##### SGLT-2 inhibitors

There is insufficient evidence to recommend the routine use of SGLT 2 inhibitors in CYP [13–16]. They can be considered for post-pubertal youth not achieving adequate control with licensed medications in collaboration with adult diabetologists.

There is a risk of euglycaemic diabetic ketoacidosis(DKA) and patients should be counselled on the symptoms and advised to seek immediate medical advice if these develop. Baseline C-peptide should be measured in those not on insulin to ensure adequate endogenous insulin production to protect against DKA. Treatment should be discontinued if DKA is suspected or confirmed and not restarted unless the cause of DKA is proven to be unrelated.

##### Other agents

There was insufficient evidence to make recommendations on sulphonylureas, DPP-4 inhibitors and orlistat (Appendix).

### Bariatric surgery

Bariatric surgery in adolescents leads to successful weight loss in those with severe obesity (BMI > 35 kg/m^2^) and demonstrates greater weight loss outcomes when compared with lifestyle and liraglutide [17].

Bariatric surgery is the most effective current treatment available for reducing metabolic comorbidities in adolescents with remission rates of 85% for type 2 diabetes, 85% for hypertension, 75% for dyslipidaemia and 78% for musculoskeletal problems [18]. Young people are more likely to have diabetes remission than adults (86%) despite having similar weight loss [19].

Nutritional deficiencies are common in adolescents with up to 70% exhibiting some form of micronutrient deficiency [18].

The impact on pregnancy in adults is mixed, with some obesity-related problems improved and others worsened [20, 21].

### Complications and comorbidities

Glycaemic control improves microvascular risk. However, the increased risk of a major cardiovascular event (MACE) in adults is not reduced by improved glycaemic control. Additional measures including reduction in excess adiposity, smoking prevention, increased physical activity and reduction of hypertension and dyslipidaemia are essential to reduce MACE risk.

### Hypertension in youth with type 2 diabetes

Treatment of hypertension in adults improves microvascular and macrovascular outcomes at least as much as improvement in glycaemic control [41]. It would be reasonable to suggest a similar improvement in outcomes could be achieved in CYP.

Blood pressure in children should be interpreted in relation to age, sex and height using appropriate centile charts [22]. Hypertension in children under 13 years of age is defined as a systolic and/or diastolic blood pressure that is greater than the 95th centile on three or more occasions and > 130/80 in those over 13 [23]. Ambulatory blood pressure monitoring can assist in the diagnosis and exclude those with white coat hypertension.

Weight loss, salt reduction and increased physical activity can improve blood pressure. ACE inhibitors should be used where blood pressure is not responsive to lifestyle change. Electrolytes should be measured at baseline and 4–6 weeks and adequate contraception is in place to avoid teratogenic side-effects.

### Dyslipidaemia

Dyslipidaemia is an important modifiable cardiovascular disease (CVD) risk factor. The classic lipid profile seen with obesity, insulin resistance and type 2 diabetes is raised triglycerides and decreased high-density lipoprotein cholesterol (HDL-C) levels. Earlier identification and control of dyslipidaemia reduces the risk of atherosclerosis in early adult life. Treatment of children with familial hypercholesterolaemia shows a reduction in subclinical atherosclerosis [51].

Dietary changes can improve dyslipidaemia and should be attempted for 6 months. Statins should be initiated where LDL-cholesterol remains > 3.4 mmol/L. Liver enzymes and CK should be monitored to detect rare liver side-effects and avoidance of pregnancy with adequate contraception is advised to avoid teratogenic side-effects. Fibrates should be used to avoid pancreatitis where triglyceride levels are very elevated.

### Non-alcoholic fatty liver disease (NAFLD)

NAFLD is a histopathological spectrum of liver disease, from early benign steatosis to non-alcoholic steatohepatitis (NASH) and cirrhosis. It contributes to the development of T2D, cardiovascular disease and chronic kidney disease [24]. T2D is one of the strongest clinical predictors of disease progression [25–27]. Steatosis is present in 25% to 50% of adolescents with type 2 diabetes and more advanced disease is increasingly common.

Screening method choice is controversial, and all are poor at differentiating disease severity. Liver enzymes should be tested at baseline and yearly thereafter. Abnormal liver function tests (LFTs) should not be attributed to NAFLD without exclusion of alternative diagnoses. Alanine transaminase (ALT) can be normal in severe disease and clinicians should have a high index of suspicion [28, 29]. Referral to a hepatologist or gastroenterologist is recommended where liver enzymes remain over 3 times the upper limit of normal despite the treatments listed below [28, 29].

Ultrasound should be performed at diagnosis and up to 3-yearly thereafter depending on risk factors. Ultrasound can identify steatosis but doesn’t adequately identify the fibrosis associated with severe disease.

Improvements in weight, diet, physical activity, insulin resistance and obstructive sleep apnoea all improve biomarkers and disease severity. Optimising vitamin D may minimise fibrosis [30]. There is insufficient evidence to recommend any one dietary approach, with limited benefit seen with low fructose, low fat and low glycaemic index diets [29, 70–73]. Psychological support (as part of multidisciplinary management) may help to improve clinical outcomes [74].

There is currently no specific pharmacological treatment for paediatric NAFLD. Metformin, vitamin E, antioxidants, fatty acid supplements and probiotics show no clear benefit on histologic outcomes or sustained reduction in ALT in CYP. In adults, pioglitazone has been recommended for advanced liver fibrosis [33–36, 75–80].

### Retinopathy

Diabetic retinopathy (DR) is a major cause of visual loss in working age adults and increases the risk of cataracts and glaucoma [37, 81, 82]. It is mainly associated with long-duration diabetes [83–85]. TODAY study showed that high HbA1c was associated with an increased risk of developing retinopathy regardless of disease duration [86].

At 20 years post diagnosis nearly all UK adults with type 1 diabetes and 60% of adults with type 2 diabetes have some degree of retinopathy [87].

Severe disease is unlikely before 12 years [37, 88] and UK guidelines recommend screening from this age regardless of disease duration.

### Albuminuria and chronic kidney disease

Kidney disease is an important complication of diabetes and is one of the most common causes of chronic kidney disease (CKD). It is characterised by persistently raised albuminuria beyond 3 months with a low estimated glomerular filtration rate (eGFR) [89].

Albuminuria is strongly associated with the progression of CKD and cardiovascular morbidity and mortality [90–93]. Albuminuria occurs more commonly in type 2 than type 1 diabetes and is likely independent of differences in body mass index and hypertension [2].

A random urine sample is the preferred outpatient screening and correlates with 24-h collection [94, 95]. Micral (dipstick) is the easiest and most cost-effective screening test [96]. An early morning sample should be tested where random urine albumin creatinine ratio (ACR) is > 3 mg/mmol [97] to minimise false-positive results due to hyperglycaemia, exercise, smoking, menstruation, recent intercourse or sample contamination [46]. Urine ACR should be raised on two of three consecutive tests obtained on different days within a 3- to 6-month period before the diagnosis is confirmed [46].

The severity of the disease should be characterised using increased ACR (3–30 mg/mmol) and severely increased ACR (> 30 mg/mmol) rather than the terms microalbuminuria and macroalbuminuria [98].

Other causes of CKD should be considered, especially if there is reduced eGRF without albuminuria or the presence of both retinopathy and albuminuria > 30 mg/mmol creatinine.

Improved blood glucose and blood pressure prevent and slow the progression of nephropathy. Therapeutic options include the use of angiotensin-converting enzyme (ACE) inhibitors or angiotensin receptor blockers (see the “Hypertension” section) [31, 32, 46, 99, 100].

We recommend referral to specialist renal/kidney care services where GFR < 30 ml/min/1.73 m^2^, consistent significant albuminuria > 30 mg/mmol, and significant haematuria or structural abnormalities are detected on ultrasound (1B) [46, 89].

### Obstructive sleep apnoea (OSA)

Obstructive sleep apnoea (OSA) is a sleep disorder characterized by repetitive episodes of upper-airway obstruction which result in intermittent hypoxemia and transient arousals leading to sleep fragmentation and poor sleep quality. Sleep disturbance and OSA are increasingly recognised as being associated with obesity, insulin resistance in adults and children and type 2 diabetes in adults. Additionally, it is a risk factor for future cardiovascular disease [101–107].

Obesity, male sex, and advancing age are the strongest risk factors for OSA [108, 109]. It affects an estimated 1–2% of normal children [110, 111] and is more common in obese youth [45].

Evaluate symptoms of obstructive sleep apnoea using questions about snoring, apnoea, nocturia, enuresis, sleep quality, morning headaches and daytime sleepiness at every visit after diagnosis [45, 112]. Questionnaires alone do not provide a high enough sensitivity or specificity and clinicians should have a high index of suspicion [113, 114].

The gold standard diagnostic test is overnight laboratory polysomnography, which quantifies episodes of apnoea and hypopnoea, with an apnoea–hypopnoea index (AHI) of > 5 being diagnostic [103]. Treatment with continuous airway pressure has been associated with improvement in the glycaemic profile, HbA1c, insulin sensitivity and inflammation in some studies [115–119].

## Conclusions

This guideline is the first national guideline on managing type 2 diabetes in children and young people. It gives practical advice on managing this challenging and aggressive condition. Paediatricians do not have extensive experience in managing type 2 diabetes due to a limited number of patients so can struggle with confidence in managing these patients. The authors hope that this guideline will provide paediatricians with the necessary information about managing type 2 diabetes and associated complications.

## Data Availability

This document is based on an evidence-based guideline. A literature search was conducted on all relevant topics and the paper summarises the main recommendations from A practical approach to Management of type 2 diabetes in CYP under18 years.

## Appendix

### Patient information leaflet

#### SGLT2 inhibitors and diabetic ketoacidosis in type-2 diabetes

**Why have I been given this leaflet?**

You are taking, or about to take, one of the following drugs for improving your diabetes management:

- Empagliflozin (Jardiance)
- Canagliflozin (Invokana)
- Dapagliflozin (Forxiga)

**What do I need to know about these drugs?**

These medications have been given to you to help you improve your weight and your blood glucose levels. However, people taking this drug can develop an unusual complication of diabetes, called euglycaemic diabetic ketoacidosis. This happens when too much acid builds up in the blood and can happen even when your blood glucose level is normal. If not identified early, this can be dangerous. However, this is a VERY RARE complication.

**What should I look out for?**

If you are taking one of these tablets, please look out for these symptoms:

- Nausea and/or vomiting,
- Fast breathing,
- Abdominal pains,
- Unusual drowsiness or
- Fever.

If you have any of these symptoms, please measure your blood ketones, if you have a blood ketone meter at home. If your levels are over 0.6 mmol/L, please contact your diabetes team immediately, even if your blood glucose is near normal. If you cannot get hold of your diabetes team, please call NHS Direct at 111 for more advice. Inform them that you are worried about “Diabetic keto-acid-osis”.

If you do not have a blood ketone meter at home, please contact your diabetes team or 111, as above.

#### Stop your medication till further medical advice

**What can cause this problem?**

This problem can develop at any time. You need to be especially careful:

- if you develop an infection (like a chest or urine infection) or undergo surgery.
- If you are planning to have an operation or any other procedure which involves fasting overnight, please discuss this medication with your doctor or nurse – you may need to stop your tablets.

**If I feel unwell, what will my doctor or nurse do?**

You will have a finger prick blood test to test for the amount of glucose and ketones (a breakdown product of fat) in the blood. If the levels of ketones are high, you may need to attend the hospital to be treated. In the meantime, please ensure you are keeping yourself well-hydrated. Please continue to take your insulin, if you usually take insulin.

## Abbreviations

ABCD: Association of British Clinical Diabetologists
ACDC: Association of Children’s Diabetes Clinicians
ACE: Angiotensin-converting enzyme
ACR: Albumin creatinine ratio
AHI: Apnoea–hypopnoea index
ALT: Alanine transaminase
BMI: Body mass index
BSPED: British Society of Paediatric Endocrinology and Diabetes
CGM: Continuous glucose monitoring
CK: Creatine kinase
CKD: Chronic kidney disease
CVD: Cardiovascular disease
CYP: Children and young people
DKA: Diabetic ketoacidosis
DPP4: Dipeptidyl peptidase
DR: Diabetic retinopathy
eGFR: Estimated glomerular filtration rate
FGM: Flash glucose monitoring
GRADE: Grading Of Recommendations, Assessment, Development and Evaluation
HDL-C: High-density lipoproteins-cholesterol
LDL: Low-density lipoprotein
LFTs: Liver function tests
MACE: Major cardiovascular event
MDT: Multidisciplinary team sodium-glucose cotransporter-2
MHRA: Medicines and health products regulatory agency
NAFLD: Non-alcoholic fatty liver disease
NASH: Non-alcoholic steatohepatitis
OSA: Obstructive sleep apnoea
Paeds QL: Paediatric quality of life
PCOS: Polycystic ovary syndrome
QOL: Quality of life
SGLT-2: Sodium-glucose cotransporter-2
SMBG: Self-monitoring of blood glucose
T2DM: Type 2 diabetes mellitus
TSDE: TODAY Standard Education Diabetes Education
